# Remarkable diastereomeric effect on thermoresponsive behavior of polyurethane based on lysine and tartrate ester derivatives

**DOI:** 10.1039/d1ra05877k

**Published:** 2021-11-03

**Authors:** Daisuke Aoki, Akihiro Miyake, Wanpen Tachaboonyakiat, Hiroharu Ajiro

**Affiliations:** Division of Materials Science, Graduate School of Science and Technology, Nara Institute of Science and Technology 8916-5 Takayama-cho Ikoma Nara 630-0192 Japan ajiro@ms.naist.jp; Department of Materials Science, Faculty of Science, Chulalongkorn University Phayathai, Pathumwan Bangkok 10330 Thailand Wanpen.Ta@chula.ac.th; Data Science Center, Nara Institute of Science and Technology 8916-5 Takayama-cho Ikoma Nara 630-0192 Japan

## Abstract

This study describes the long-distance diastereomeric effect on thermoresponsive properties in water-soluble diastereomeric polyurethanes (PUs) composed of an l-lysine ethyl ester diisocyanate and a trimethylene glycol l-/d-tartrate ester, which have differences in spatial arrangements of the ethyl esters in the mirror image. The PUs based on l-lysine and l-/d-tartrate ester, named l-PU and d-PU, were synthesized with various number average molecular weights from 4700 to 13 100. In turbidimetry, l-PU showed a steep phase transition from 100%*T* to 0%*T* within about 10 °C at 4 g L^−1^, whereas d-PU did not change completely to 0%*T* transmittance even at 80 °C at 4 g L^−1^. In addition, the thermoresponsive properties of l-PU were less affected by concentration than those of d-PU. This long-distance diastereomeric effect on thermoresponsive behavior between l-PU and d-PU appeared in common among 6 samples with 4700 to 13 100 number average molecular weight. In the dynamic light scattering experiments at each transmittance, the hydrodynamic diameter (*D*_h_) of l-PU increased up to 1000 nm, while the *D*_h_ of d-PU remained almost at 200–300 nm. The C=O stretching vibration of FT-IR spectra showed that d-PU has more hydrogen-bonded ester groups than L-PU. Thus, we speculated that the difference in the retention of polymer chains in the micelle to promote intermicellar bridging generates the long-distance diastereomeric effect.

## Abstract 2

The long-distance diastereomeric effect on thermoresponsive properties in a polyurethane system consisting of chiral monomers was reported.

## Graphical abstract

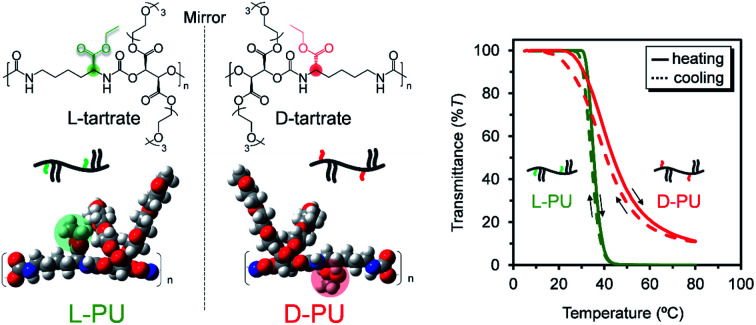

## 1. Introduction

Thermoresponsive polymers have been developed in a wide range of molecular designs because of their potential application as smart materials.^1–4^ Stereoregularity is a helpful molecular design strategy for controlling thermoresponsive properties using conformational entropy. In nature, proteins can form their precise folding structure by controlling their conformations using the stereoregularity of an l-amino acid. Interestingly, the change of a particular amino acid residue from the l-form to the d-form sometimes leads to amyloid fibril formation and inhibition.^5^ This long-distance diastereomeric effect along polymer chains inspires a new platform for thermoresponsive polymer design because a small amount of chirality reversal is amplified into macroscopic change.

The stereoregularity of artificial polymers has been related to their thermoresponsive properties.^6–10^ For instance, poly(*N*-isopropyl acrylamide) (PNIPAm) shows different thermoresponsive behavior through its tacticity. It has been reported that syndiotactic rich PNIPAm exhibits sharp phase transition behavior due to the enhancement of cooperative hydration.^7^ In contrast, isotacticity of PNIPAm leads to a broader phase transition because of increasingly strong hydrogen bonding interaction within polymer chains.^8,9^ This different behavior in water media mostly depends on whether the hydrogen bonding of amide side chains strongly interacts with water molecules or another amide side chain.^7,9,10^ On the other hand, poly oligo(ethylene glycol) (OEG) methacrylate derivatives exhibit no stereoregular effect on thermoresponsive behavior except for a small difference in cloud point (*T*_cp_).^11^ Furthermore, stereoregular polyether bearing OEG at the side chains also acts similarly in water media to an atactic polymer.^12^ Thus, the absence of strong intramolecular hydrogen bonds may desensitize the stereoregular effect despite the close distance between asymmetric carbon atoms. In addition, amino acid-based polymers have been associated with thermoresponsive helical conformation in water.^13–19^ Only a few studies of amino acid-based polymers have reported a macroscopic difference in turbidity due to their chirality.^18,19^ In short, studies on the thermoresponsive behavior in stereoregular polymers have been focused on the hydrogen bond and polymer systems based on close asymmetric atoms. The octadiene backbone polymer has been reported to exhibit a slightly different *T*_cp_ by the regio- and stereo-regularity in an example associated with the long-distance diastereomeric effect.^20^ However, the long-distance diastereomeric effect was not the focus of the study.

Therefore, stereoregular thermoresponsive polymers with a long distance between asymmetric carbons are an undeveloped area, and are important in deepening our understanding of thermoresponsive behavior. In this context, we previously reported thermoresponsive polyurethane (PU) consisting of hexamethylene diisocyanate (HDI) and chiral l-tartrate ester with OEG side chains.^21^ However, the long-distance diastereomeric effect did not appear because the chiral relationship between reported PUs was composed of an achiral diisocyanate and symmetric chiral l-/d-tartrate diols.

**Fig. 1:**
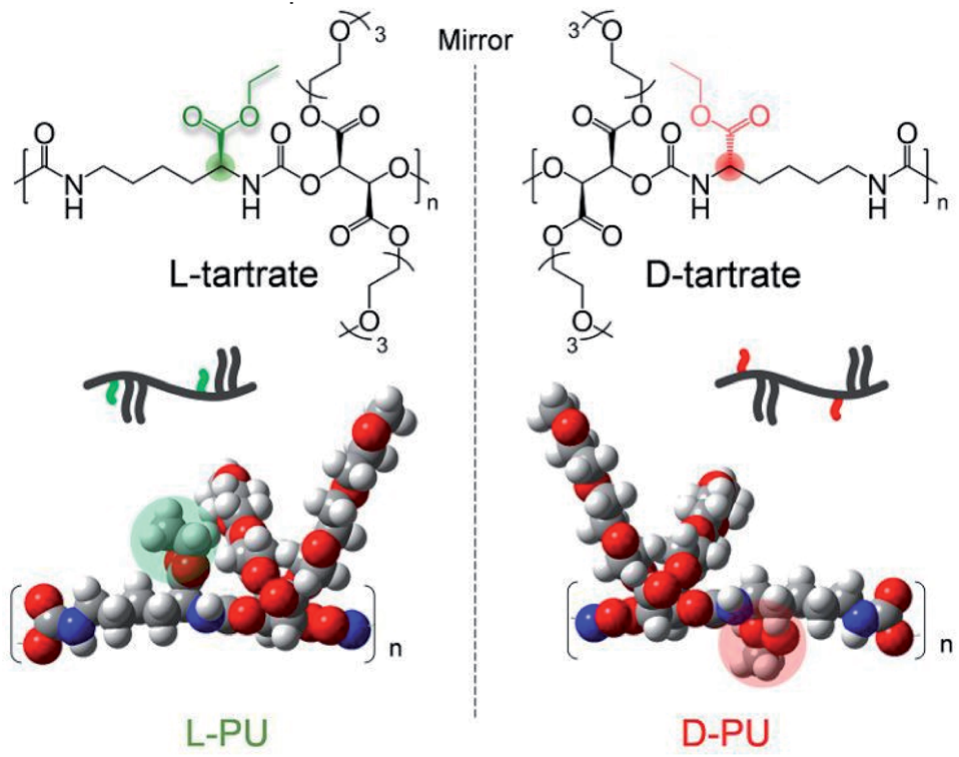
Chemical structure of diastereomeric water-soluble polyurethanes, l-PU and d-PU.

In this study, we propose the long-distance diastereomeric effect on thermoresponsive behavior in polyurethane diastereomers consisting of an asymmetric l-lysine-based diisocyanate (LDI) and a symmetric diol OEG l-/d-tartrate ester bearing two chiral carbons. This diastereomeric PU system is imagined as different spatial arrangements of ethyl esters in the mirror image, although the actual difference in chirality is derived from tartrates (Fig. 1). Surprisingly, the slight spatial difference of the long-range ethyl ester groups was amplified, leading to different thermoresponsive behavior in water media.

## 2. Experimental

### 2.1. Materials

l-Lysine ethyl ester diisocyanate (LDI) was purchased from Alfa Aesar Co. Dibutyltin dilaurate (DBTDL), triethylene glycol monomethyl ether (mOEG_3_), *p*-toluenesulfonic acid monohydrate (TsOH) were purchased from Tokyo Chemical Industry. l-Tartaric acid and d-tartaric acid were purchased from Nacalai Tesque, Inc. Anhydrous tetrahydrofuran (THF) and anhydrous *N*,*N*-dimethylformamide (DMF) were purchased from Kanto Chemical Co., Inc. Diethyl ether and benzene were purchase from Wako Pure Chemical Industries. The chiral diol monomer, l-OEG_3_TA and d-OEG_3_TA, were synthesized following the literature procedure^21^ by condensation between tartaric acid and mOEG_3_ using TsOH as an acid catalyst.

### 2.2. Polymerization for l-PU and d-PU

The general procedure was conducted according to our previous report.^20^ OEG_3_TA (1.77 g, 4 mmol) was dried in a 20 mL vial equipped with septum rubber at 80 °C overnight. Next, the vial was filled with N_2_ gas and injected anhydrous solvent (THF or DMF), DBTDL (1 drop), and LDI (0.8 mL, 4 mmol) with the syringe. After the mixture was stirred at 50 °C for 8 h, the polymer was precipitated into an excess amount of diethyl ether. Finally, the resultant precipitated polymer was dried *in vacuo* at 60 °C.

### 2.3. Measurements

^1^H NMR spectra were measured using a JEOL JNM-ECX400 apparatus. The Fourier transform infrared spectrometry (FT-IR) spectra were measured using a Shimadzu IRAffinity-1S spectrometer. Size-exclusion chromatography (SEC) was performed using a JASCO LC-2000 Plus series system equipped with a PU-2080 Plus Intelligent HPLC pump, a RI-2031 Plus Intelligent RI detector, a CO-2065 Plus Intelligent column oven, an AS-2055 Plus Intelligent Sampler, and two linearly connected commercial column (TSKgel Super3000 and GMHXL, Tosoh Corporation) using THF as an eluent at 40 °C at a flow rate of 0.1 mL min^−1^. The molecular weight calibration curve was calculated using polystyrene standard (Shodex). Differential Scanning Calorimetry (DSC) was performed using Hitachi DSC6200 at a rate of 2 °C min^−1^ for polymer aqueous solution (1/1, w/w). UV-vis transmittance curves for polymer aqueous solution was obtained on UV-2600 (Shimadzu) system at 500 nm with heating and cooling from 10 to 80 °C at a rate of 1 °C min^−1^. The cloud point temperature (*T*_cp_) was defined as temperature at 50% UV-vis transmittance. Dynamic light scattering (DLS) measurements were performed on a Malvern ZEN 3600 Zetasizer Nano ZS equipped with a monochromatic coherent He–Ne laser with a fixed wavelength of 633 nm and 20 °C.

## 3. Results and discussion

### 3.1. Synthesis and characterization of diastereomeric PUs

**Scheme 1:**
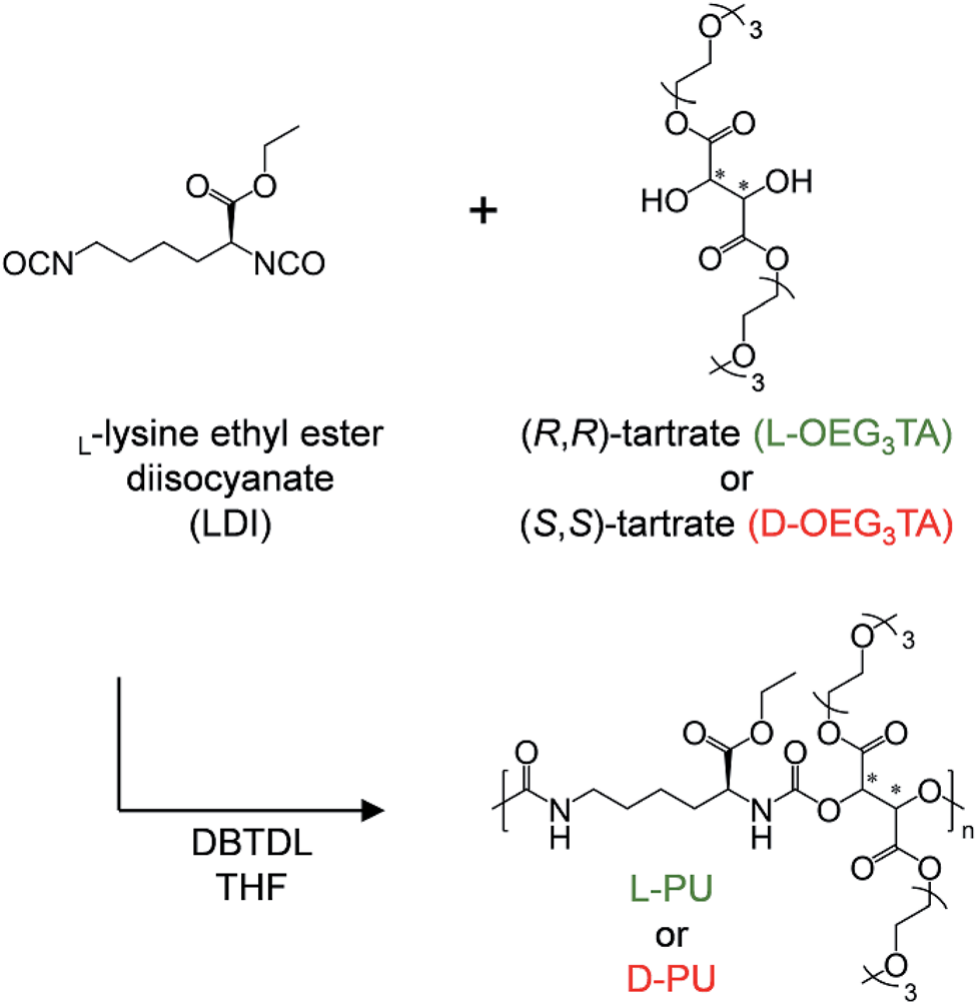
Synthesis of l-PU and d-PU.

We designed and synthesized the diastereomeric water-soluble PUs by polyaddition between commercially available l-lysine diisocyanate ethyl ester (LDI) and one-step synthetic three units of OEG l-/d-tartrate ester diol monomer (OEG_3_TA) (Scheme 1). The previous PUs composed of HDI and OEG3TA were difficult to control polymerization, with a number average molecular weight (*M*_n_) up to 6500 and a large *M*_w_/*M*_n_ of about 4.^21^ We attributed this problem to OEG_3_TA impurity mixing a small amount of mOEG_3_ and a low reaction rate and addressed it by changing the eluent of column chromatography and extending the polymerization time.

In addition, it is noteworthy that l-lysine and tartaric acid are an abundant chiral source and have low toxicity due to their natural origin. This novel chiral PU system is the diastereomeric relationship derived from only the ethyl ester spatial position under the mirror image. According to the Ramachandran plot, this slight spatial position difference probably amplifies the conformational behavior because the lysine residue prefers twisted dihedral angles are prone to form a helix structure.^22^

**Fig. 2:**
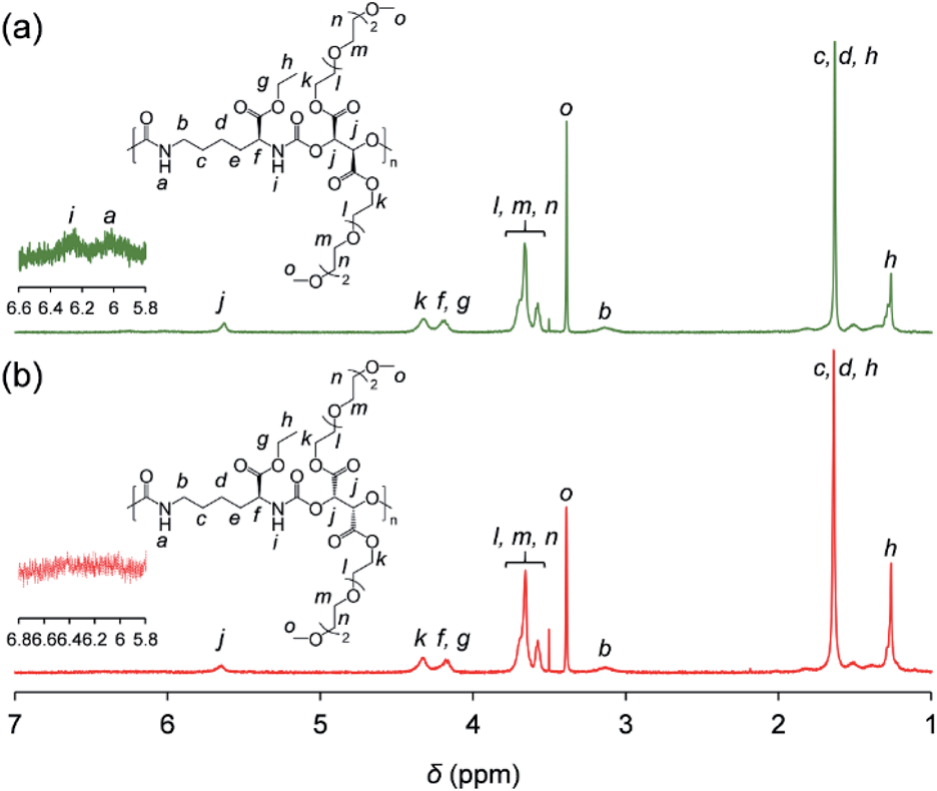
^1^H NMR (400 MHz) spectra of (a) l-PU and (b) d-PU in CDCl_3_.

**Fig. 3:**
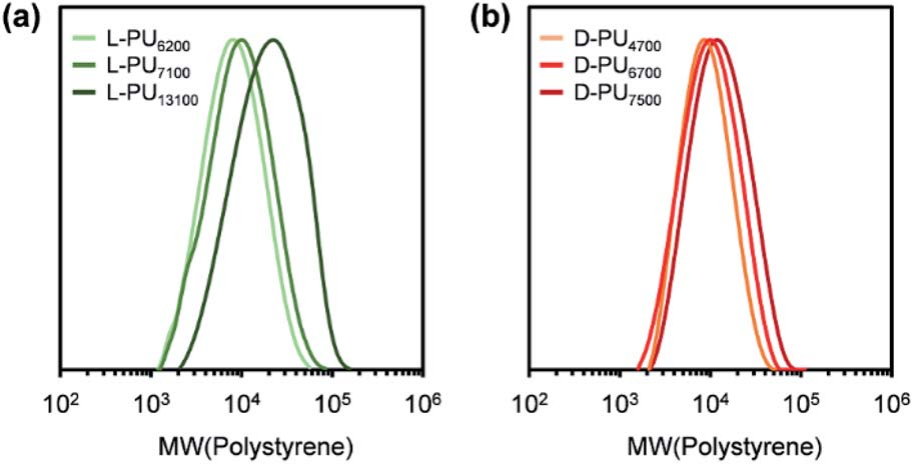
SEC profiles for l-PU and d-PU with polystyrene equivalent using THF as an eluent.

Synthesized PUs based on l-/d-tartrate ester, named l-PU and d-PU, were characterized by ^1^H NMR, SEC, and FT-IR. Fig. 2 shows ^1^H NMR spectra of l-PU and d-PU. As to our previous reports,^21^ the methine proton peak j adjacent to the alcohol group in OEG_3_TA shifted from 4.7 ppm to 5.7 ppm with the formation of urethane bonds. There was no noticeable spectral difference between l-PU and d-PU because of the chemical compositional equivalent (Fig. 2a and b). In addition, since end group peaks observed in the previous work were not detected, the resultant PUs were successfully polymerized with high *M*_n_ than the previous OEG_3_TA based PU. Fig. 3 shows the SEC profiles with polystyrene equivalent for l-PU and d-PU. Polymerization was successfully controlled, as the *M*_w_/*M*_n_ and *M*_n_ were about 2 for all PUs and up to 13 100. We polymerized these diastereomeric PUs with a different *M*_n_, ranging from 4700 to 13 100 to highlight the diastereomeric effect on thermoresponsive behavior. In the following discussion, we compared PUs with almost the same average molecular weight and dispersity samples (l-PU: *M*_n_ = 7,100, *M*_w_/*M*_n_ = 1.76 and d-PU: *M*_n_ = 7,500, *M*_w_/*M*_n_ = 1.49).

**Fig. 4:**
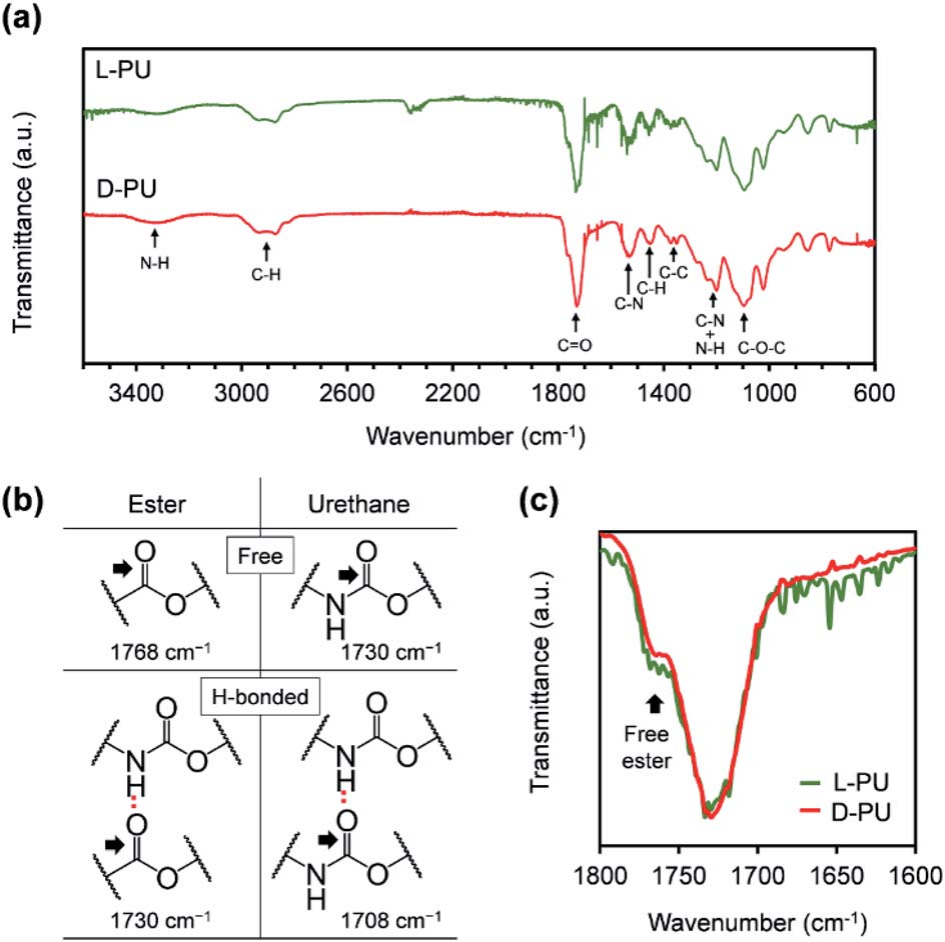
(a) FT-IR spectra for l-PU and d-PU. (b) Assignment of the C=O stretching vibration. (c) Enlarged spectra showing the C=O stretching vibration region for l-PU and d-PU.

Next, we characterized FT-IR spectra for l-PU and d-PU to confirm the formation of urethane bonds and their hydrogen bonding (Fig. 4a).^23–27^ Both l-PU and d-PU exhibited the N–H and C=O stretching vibration peaks around 3300 and 1730 cm^−1^ in FT-IR spectra, suggesting the urethane bond formation. In addition, the FT-IR peaks of the ether C–O–C bonds in the OEG chains the urethane C–N bonds supported the successful synthesis of the desired PUs bearing OEG side chains. Furthermore, we attributed the C=O stretching vibration peaks to the free and hydrogen-bonded ester bonds at around 1768 and 1730 cm^−1^, free and hydrogen-bonded urethane bonds at around 1730 and 1708 cm^−1^, according to the literature (Fig. 4b).^25–27^ The broad peak at around 1730 cm^−1^ probably contains the hydrogen-bonded ester C=O and the free urethane stretching vibration with slightly different peak tops. We considered the urethane hydrogen bonding by the area of the C=O stretching vibration. Both l-PU and d-PU showed the small area of the hydrogen-bonded urethane C=O stretching vibration peaks (<10%), which are much lower than that of a series of our previous HMDI-based PUs.^27^ This is because the steric repulsion of ethyl and OEG ester groups on the carbon adjacent to the urethane bond prevents hydrogen bonding between the urethane bonds. However, though the C=O stretching vibration peaks of l-PU and d-PU were almost identical, d-PU showed a slightly smaller free ester bond shoulder proportion than l-PU (Fig. 4c). Considering that the *M*_n_ of l-PU and d-PU is comparable, the ratio of urethane bonds to ester bonds should match between l-PU and d-PU. Thus, the difference in the proportion of the free ester C=O stretching vibration at 1768 cm^−1^ is caused by shifting to the hydrogen-bonded ester C=O stretching vibration. In other words, d-PU has more hydrogen-bonded ester bonds. This reflects the difference in interaction based on the ethyl ester groups' spatial arrangement in mirror image, leading to a unique diastereomeric effect on thermoresponsive properties.

### 3.2. Thermoresponsive behavior of diastereomeric PUs

**Fig. 5:**
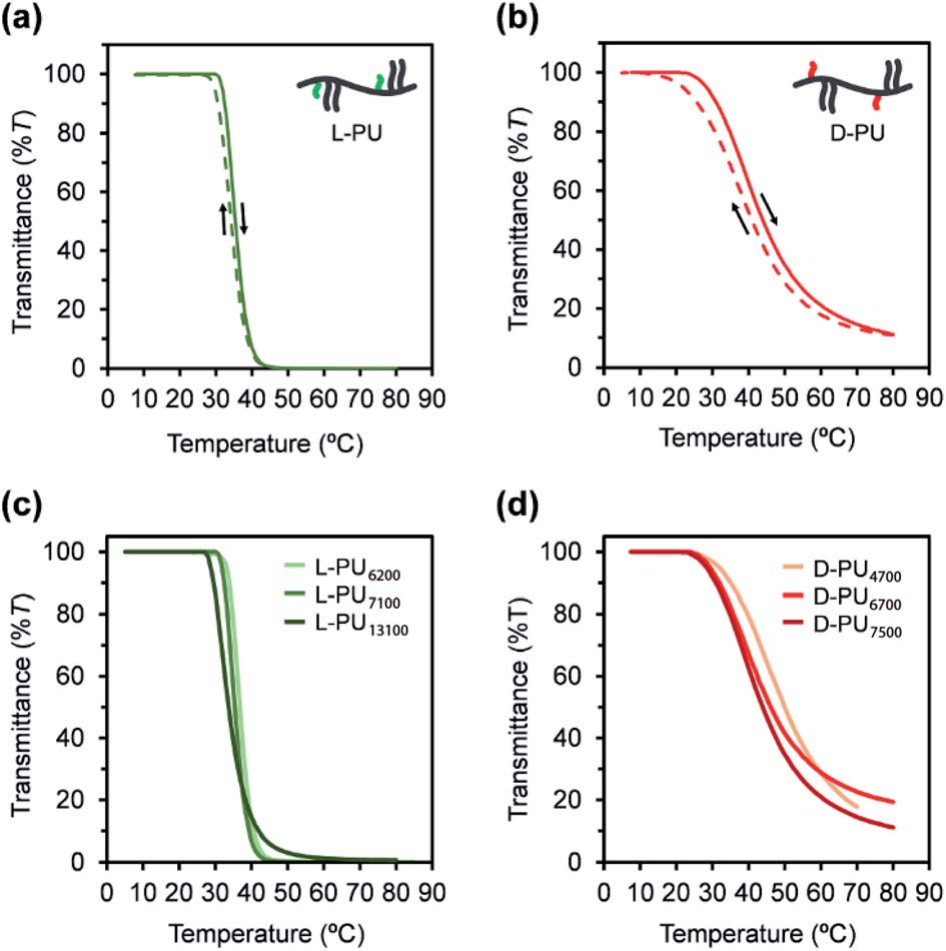
UV-vis transmittance curves for the (a) l-PU and (b) d-PU (*M*_n_ ∼7000) with 2 g L^−1^ aqueous solution in heating cycle (solid line) and cooling cycle (dotted line). UV-vis transmittance curves for aqueous solution of (c) l-PU and (d) d-PU with different *M*_n_.

First, we compared the turbidimetry of l-PU and d-PU. Fig. 5a and b show UV-vis transmittance curves of 500 nm light for l-PU and d-PU in 2 g L^−1^ aqueous solution from 10 to 80 °C at a rate of 1 °C min^−1^. Surprisingly, we observed an entirely different thermoresponsive behavior between the l-PU and d-PU diastereomers. The transmittance curve of the l-PU was steep in the range of 5–10 °C, comparable to that of well-studied typical thermoresponsive polymers, including PNIPAm and POEGMA.^28^ On the other hand, the d-PU showed a gentle transmittance curve, not to reach 0%*T* at even 80 °C. We observed the same tendency in the steepness of l-PU and d-PU transmittance curves in all samples with different *M*_n_ (Fig. 5c and d).

**Fig. 6:**
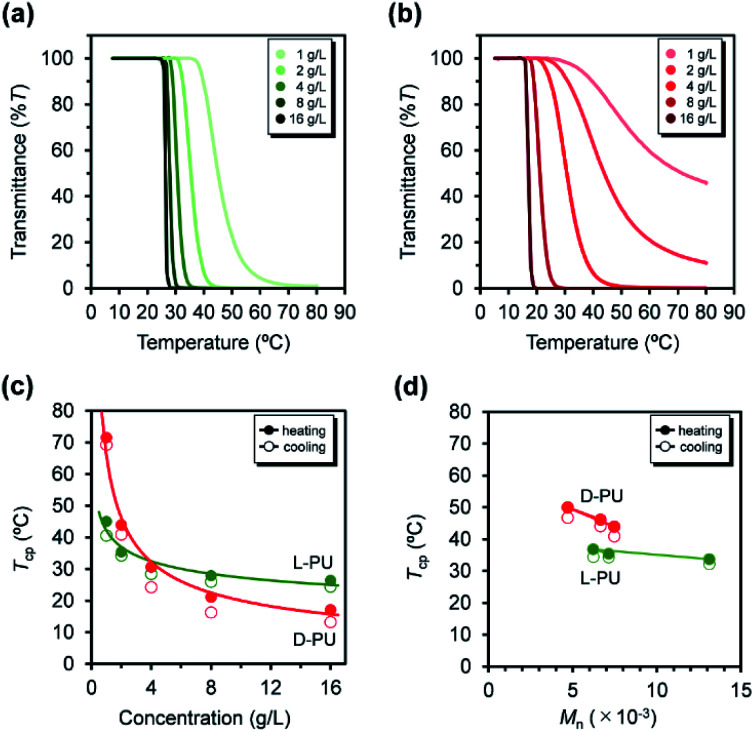
UV-vis transmittance curves at various concentrations from 1 to 16 g L^−1^ for (a) l-PU and (b) d-PU. Plots of the measured temperature at cloud point (*T*_cp_) for l-PU and d-PU as a function of (c) polymer concentration in water and (d) the number average molecular weight (*M*_n_) of polymer aqueous solution at 2 g L^−1^ (solid line, heating; dotted line, cooling).

In addition, we investigated the concentration effect from 1 g L^−1^ to 16 g L^−1^ on UV-vis transmittance of l-PU and d-PU in aqueous solutions during the heating process (Fig. 6a and b). Both l-PU and d-PU showed a similar steep phase transition at the high concentration, and their cloud point (*T*_cp_) was 28 °C and 19 °C at 16 g L^−1^. However, for the dilute region, l-PU still showed steep transition curves at up to 2 g L^−1^, whereas d-PU lost steepness below 4 g L^−1^. This apparent difference in the transmittance curves depending on concentration is related to the polymer agglutination process. We compared the *T*_cp_ plots for each concentration of l-PU and d-PU (Fig. 6c). The phase boundary curve of d-PU decreased more steeply with increasing concentration than the l-PU counterpart. However, the decaying decrease in the l-PU and d-PU phase boundary curves differed from the stereoregular PNIPAm. According to the literature, the meso-rich PNIPAm consisting of 60% of meso carbon showed an almost plateau phase boundary curve, whereas the 46% counterpart showed a monotonically decreasing curve with concentration.^6,9,29^ Here, the monotonic decrease of *T*_cp_ with concentration is associated with a cooperative hydration structure leading to a steep UV-vis transmittance curve. In short, the plateau phase boundary curve of only meso rich PNIPAm tends to result in broad UV-vis transmittance curves. However, when the comparison is only above the 10 g L^−1^ region, the boundary curve of d-PU is a plateau rather than monotonically decreasing, suggesting that both l-PU and d-PU do not form cooperative hydration. Therefore, the difference in thermoresponsive behavior between the l-PU and d-PU may not be due to the cooperative hydrogen bond. Furthermore, to generalize the diastereomeric effect between l-PU and d-PU, we investigated the molecular weight effect, which influences thermoresponsive behavior. Table 1 summarized the *T*_cp_ of different *M*_n_ samples from 6200 to 13 100 for l-PU and from 4700 to 7500 for d-PU with *M*_w_/*M*_n_ controlled around 2. Fig. 6d shows plots of *T*_cp_ as a function of *M*_n_. The *T*_cp_ for l-PU and d-PU does not overlap on the plots. Moreover, the molecular weight dependence of d-PU was more significant than l-PU, suggesting that d-PU is affected by the end group and particle size.

**Table 1:** Summary of thermoresponsive properties for chiral polyurethanes

Entry	Sample	*M*_n_ a (×10^−3^)	*M*_w_/*M*_n_	*T*_cp_ b (°C)
A	l-PU_6200_	6.2	1.64	37
B	l-PU_7100_	7.1	1.76	35
C	l-PU_13100_	13.1	1.64	34
D	d-PU_4700_	4.7	1.98	50
E	d-PU_6700_	6.7	2.16	46
F	d-PU_7500_	7.5	1.58	44

a Determined by SED based on PS standard, eluted by THF.

b Determined by 50% transmittance for 2 g L^−1^ polymer aqueous solution on UV-vis transmittance curves at a heating rate of 1 °C min^−1^.

**Fig. 7:**
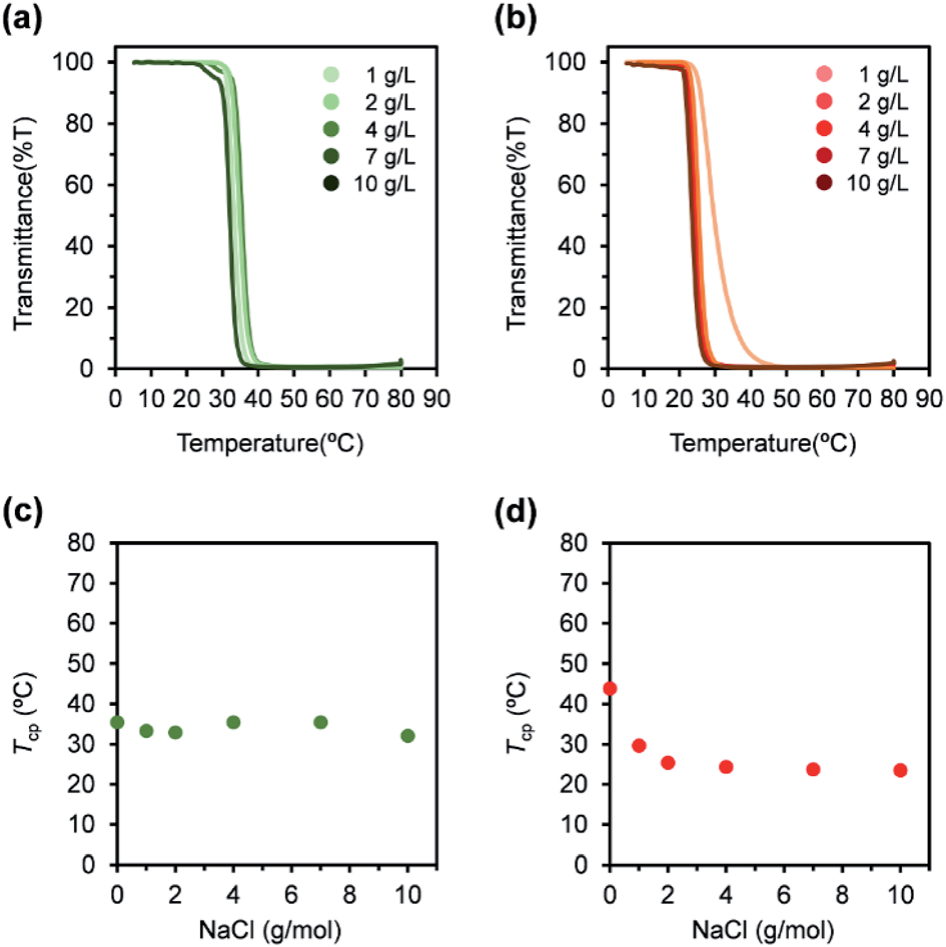
UV-vis transmittance curves for (a) l-PU and (b) d-PU with different NaCl concentration. Plots of the measured temperature at cloud point (*T*_cp_) for (c) l-PU and (d) d-PU with different NaCl concentration. Polymer concentration in water was 2 g L^−1^.

The l-PU and d-PU 2 g L^−1^ aqueous solution also showed different salting-out effects with NaCl. *T*_cp_ of l-PU gradually decreased for NaCl concentration, comparable to PNIPAm and previous thermoresponsive PUs consisting of HDI and OEG_3_TA (Fig. 7a and c).^21,28^ On the other hand, the *T*_cp_ of d-PU showed a steep decrease at low NaCl concentration and moderated as the concentration increased (Fig. 7b and d). According to the literature, the preferred cation coordination to the amide group in PNIPAm partially offsets the salting-out effect.^30^ Based on this idea, the Na^+^ cations may not reach the coordination sites of ester and urethane groups in d-PU at the low concentration, and the salting-out is not sufficiently offset. Therefore, we speculated that d-PU particles have weak inter-particle interaction due to urethane groups embedded inside.

### 3.3. Differential scanning calorimetry

**Fig. 8:**
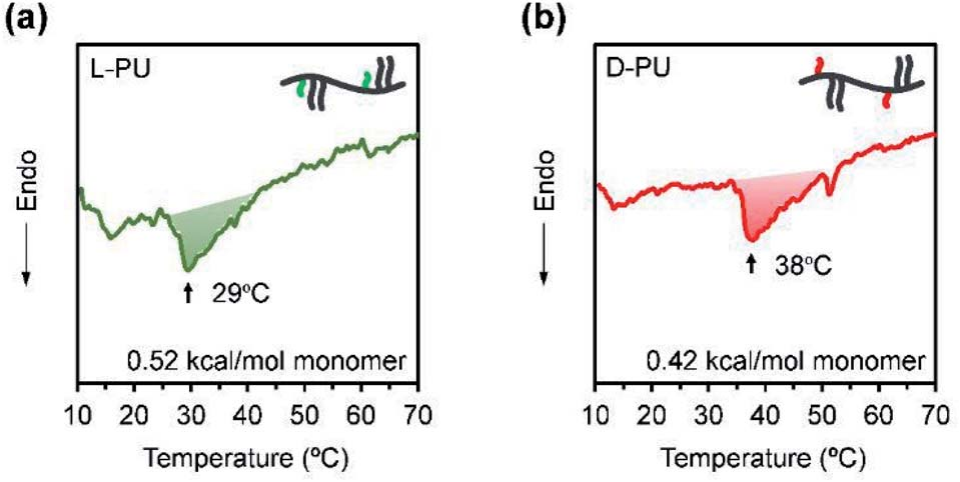
DSC curves for (a) l-PU and (b) d-PU 50 wt% aqueous solution.

Next, we considered the microscopic thermoresponsive mechanism using differential scanning calorimetry (DSC) to understand the different macroscopic turbidimetry of l-PU and d-PU. DSC reflects a phase transition behavior as an endothermic peak caused by the dehydration process surrounding thermoresponsive polymers. Fig. 8a and b show DSC curves for aqueous solutions of l-PU and d-PU. We observed broad endotherm peaks at 38 °C and 29 °C for l-PU and d-PU, respectively. The enthalpy of the monomer units along the chain was estimated to be 0.52 and 0.42 kcal mol^−1^ for l-PU and d-PU, respectively. These enthalpy values are lower than hydrogen bonding PNIPAm, typically with a 0.8–1.9 kcal mol^−1^, similar to non-hydrogen bonding POEGMA derivatives.^31–34^ Moreover, the peak shapes of l-PU and d-PU are broad and almost the same, which is identical to POEGMA.^27^ Therefore, the hydrophobicity of the OEG side chains rather than the hydrogen bonds of the urethane bonds may be dominantly involved in the aggregation process. According to the literature,^7^ the cooperative hydration of syndiotactic rich PNIPAm shows sharper endothermic peaks than isotactic rich PNIPAm. Thus, l-PU and d-PU probably do not have a cooperative hydration because of the lysine alkyl spacer. From the above, the difference in visible turbidity is not ascribe to cooperative hydration on the molecular scale as in PNIPAm.

### 3.4. Dynamic light scattering

**Fig. 9:**
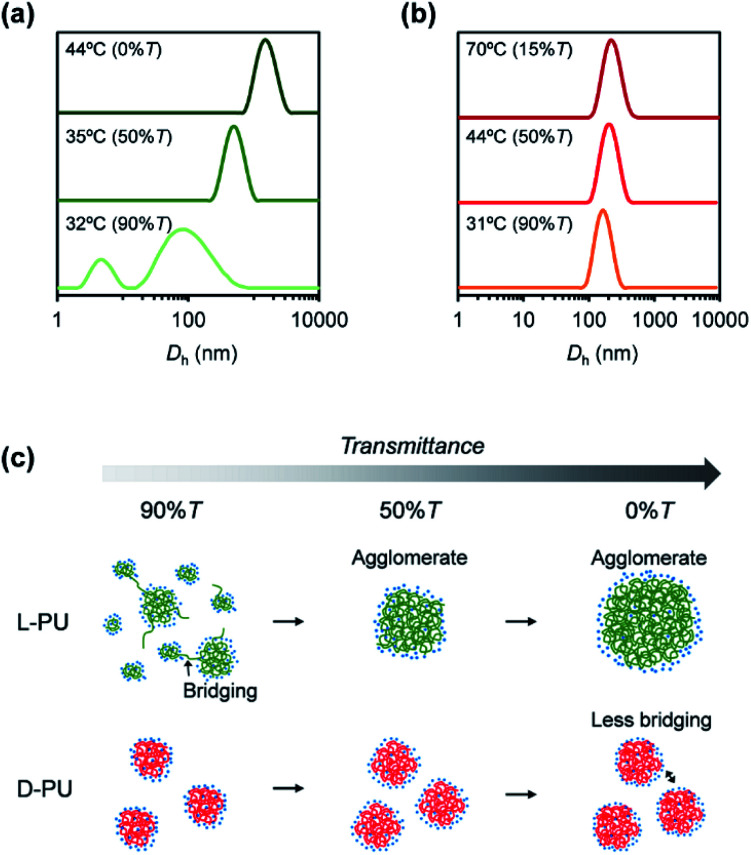
Temperature-dependent DLS traces for (a) l-PU and (b) d-PU in water at a concentration of 2 g L^−1^. (c) Schematic representation for the possible thermoresponsive mechanism of l-PU and d-PU during turbidimetry.

To clarify the aggregation process of l-PU and d-PU on the particle scale, we measured the particle size at the temperature at each transmittance (%*T* = 90, 50, 0, or 15) by dynamic light scattering (DLS). The aggregation process of l-PU is similar to traditional thermoresponsive hydrogen-bonding polymers that significantly change their hydrodynamic diameter (*D*_h_) ∼1000 nm over *T*_cp_ (Fig. 9a).^16,35^ At 32 °C, corresponding to 90%*T* of 2 g L^−1^ aqueous solutions, l-PU exhibited broad and bimodal peaks that included particles of many sizes because of insufficient particle interaction. Above 35 °C, l-PU showed a monomodal aggregate peak and gradually increased in size depending on the transmittance. On the other hand, the d-PU showed almost constant *D*_h_ ∼200 nm over a wide temperature range (Fig. 9b). d-PU showed a monomodal peak in 160 nm even at 90% transmittance whereas l-PU was bimodal. Even raising the temperature to 70 °C, the *D*_h_ of d-PU only increased to 210 nm. Retaining the *D*_h_ at high temperatures supports a strong dependence of polymer and NaCl concentration on UV-vis transmittance, which is attractive in designing colloidal stability using chirality. This stable *D*_h_ of d-PU is due to the strength of the interaction of the particles after dehydration. Indeed, polymers consisting of OEG tend to aggregate to a *D*_h_ ∼200–300 nm ceiling because the driving force is only hydrophobic interaction.^36,37^ On the basis of the results mentioned above, we propose a possible mechanism for the long-distance diastereomeric effect on thermoresponsive behavior (Fig. 9c). Here, we focused on the growth process of the formed micelles *D*_h_ ∼100 nm. The l-PU aggregates efficiently once a small micelle appears due to more vital interparticle interaction than d-PU. On the other hand, d-PU forms weak interparticle interaction particles up to the *D*_h_ ∼200 nm ceiling despite agglomerating earlier than l-PU. According to the literature,^38–40^ when a dehydrated polymer chain end leaves from a micelle by thermal motion, the chain is likely to migrate to another micelle and lead to intermicellar bridging. Given that this process is accelerated, particle size grows to form an agglomerate with a huge particle size (*D*_h_ ∼1000 nm). Thus, l-PU promoted intermicellar bridging because of more easily releasing polymer chains from the micelles, whereas d-PU kept polymer chains in the micelle and remained stable micelle size. This assumption was supported by the higher proportion of hydrogen-bonded ester of d-PU than that of l-PU in FT-IR spectra. Therefore, the slight difference in the ethyl ester group spatial arrangement was reflected in the retention of the dehydrated polymer chain in the micelle *via* hydrogen bonding to the ester group, resulting in the long-distance diastereomeric effect.

## 4. Conclusions

In conclusion, we found a long-distance diastereomeric effect on thermoresponsive behavior in chiral PUs consisting of l-lysine diisocyanate and l-/d-OEG tartrate ester diol. l-PU and d-PU displayed the clearly different thermoresponsive behavior on UV-vis transmittance despite the lysine alkyl spacer. l-PU showed steep transmittance curves (<10 °C) over a wide range of concentrations (2–16 g L^−1^), whereas d-PU was broad (>10 °C) below 4 g L^−1^ concentration. The DSC curves of l-PU and d-PU showed small endothermic peaks with almost the same enthalpy (l-PU: Δ*H* = 0.52 kcal mol^−1^ and d-PU: Δ*H* = 0.42 kcal mol^−1^), suggesting that the dehydration process is the same on the molecular scale almost without the cooperative hydration. Furthermore, DLS showed that the particle size of d-PU was nearly constant over a wide range of temperatures (31–70 °C). In contrast, l-PU significantly increased in particle size, consistent with typical thermoresponsive polymers. Thus, the interparticle interactions were altered by the slight chirality of the ethyl groups, producing differences in turbidimetry. This study is the first report of the long-distance diastereomeric effect reflected in macroscopic thermoresponsive behavior despite the flexible lysine spacer. Given the ease of introducing chirality into step-growth polymerization systems, the long-distance diastereomeric effect is expected to be a new platform for molecular design.

## Conflicts of interest

There are no conflicts to declare.

## Supplementary Material
